# Assessment of Biological Properties of Recombinant Lumpy Skin Disease Viruses with Deletions of Immunomodulatory Genes

**DOI:** 10.3390/v17101390

**Published:** 2025-10-19

**Authors:** Aisha Issabek, Arailym Bopi, Nurlan Kozhabergenov, Bermet Khudaibergenova, Kulyaisan Sultankulova, Olga Chervyakova

**Affiliations:** 1 Research Institute for Biological Safety Problems (RIBSP), National Holding QazBioPharm, Gvardeiskiy uts 080409, Kazakhstan; a.bopi@biosafety.kz (A.B.); n.kozhabergenov@biosafety.kz (N.K.); k.sultankulova@biosafety.kz (K.S.); 2 National Academy of Sciences of the Kyrgyz Republic, Bishkek 720010, Kyrgyzstan; khudaibergenbermet@gmail.com

**Keywords:** lumpy skin disease virus, immunomodulatory gene, vaccine vector

## Abstract

Rational design of capripoxvirus-based vaccine vectors can be achieved by knockout of immunomodulatory genes. In this study, the effect of knockout of the immunomodulatory genes LSDV005, LSDV008 and LSDV066 on the replication of Lumpy skin disease virus in cell cultures and the immune response to an integrated foreign antigen were assessed. The knockout of genes was performed by homologous recombination under conditions of temporary dominant selection. It was found that single knockout of the LSDV005 gene and the LSDV008 gene did not affect the replicative activity of recombinant viruses in vitro (Atyrau-5 and Atyrau-B). Both single knockout of the LSDV066 gene and in combination with knockout of LSDV005 or LSDV008 led to a decrease in the replicative activity of recombinant LSDVs. The recombinant Atyrau-5J(IL18) with LSDV005 gene knockout induced production of antibodies to the integrated antigen in mice. Prime-boost vaccination with all studied recombinants increased the level of interferon-γ. In addition, during immunization with the recombinant Atyrau-5J(IL18) secretion of interleukin-2 was significantly increased. The study of the functions of immunomodulatory genes and their effect on the expression of inserted sequences of foreign antigens is promising for the creation of highly effective polyvalent vector vaccines for animals.

## 1. Introduction

Lumpy skin disease virus (LSDV) is the causative agent of a highly contagious transboundary disease of cattle, which causes serious economic damage to livestock production. Like sheep pox virus and goat pox virus, Lumpy skin disease virus belongs to the *Capripoxvirus* genus of *Poxviridae* family.

The genome of capripoxviruses is represented by double-stranded DNA of about 150 kb, encoding up to 156 open reading frames (ORFs) [1,2]. The central part of the genome includes genes that are relatively conserved among poxviruses and perform general functions such as replication and virion assembly. More variable genes encoding proteins responsible for limiting host range and modulating the host immune response to infection are located terminally [3].

Attenuated capripoxviruses are used as live vaccines to prevent related infections [4]. Since capripoxviruses, like other poxviruses, are capable of incorporating foreign DNA into their genome, they are used as vaccine vectors for foreign gene sequences. Using capripoxviruses, bivalent vaccines have been developed against Lumpy skin disease and rabies [5], Lumpy skin disease and Rift Valley fever [6], sheeppox and peste des petits ruminants [7], Lumpy skin disease and bovine ephemeral fever [8]. The range of susceptible hosts for capripoxviruses is limited to ruminants. In the cells of other organisms, capripoxvirus infection is abortive, which makes these viruses safe and promising for use as vectors with reduced replication to develop vaccines for both animals and humans [9,10].

Knowledge about the interaction of viruses with body cells is constantly growing. However, it still remains unclear what effect the vaccine vector has on the activation of immune responses in the host through the expression of viral immunomodulatory genes. Poxviruses acquired immunomodulatory genes during coevolution with their hosts and developed various strategies to counteract the host’s immune responses [11]. Poxviruses produce proteins that mimic host receptors or cytokines, thereby actively blocking extracellular immune signals necessary for effective viral clearance [12]. Also, intracellular viral proteins disrupt communication between the infected cell and the cellular component of the immune system by slowing innate antiviral responses such as apoptosis [12]. Thus, immunomodulatory genes are involved in the induction and/or suppression of the activity of various components of the immune system [13]. Studying the influence of immunomodulatory viral genes on the formation of an immune response will contribute to the improvement of vaccine vectors to ensure the formation of a long-term effective immune response.

One of the most common strategies to produce new poxvirus vectors with reduced virulence and have higher immunogenicity is knockout of immunomodulatory genes [14]. Potential immunomodulatory genes of capripoxviruses have been identified through genome sequencing and analysis [1,2]. The aim of this study was to investigate the effect of the immunomodulatory genes LSDV005, LSDV008 and LSDV066 on the replication of Lumpy skin disease viruses in vitro and the formation of an immune response to an inserted foreign antigen.

## 2. Materials and Methods

### 2.1. Cells and Viruses

During an outbreak of Lumpy skin disease in 2016 in the Atyrau region, scientists from the Research Institute for Biological Safety Problems (RIBSP) isolated the causative agent of the infection [15]. In this study, the isolated virus Dermatitis nodulares/2016/Atyrau/KZ (Atyrau/KZ) was used to assess the effect of immunomodulatory genes on the immune response.

Lamb testicular cells (LT) were used for propagation, genome recombination, and determination of the biological activity of Lumpy skin disease viruses. African green monkey kidney (Vero) cells were used for determination of reproductive ability. Used cell cultures were presented by the Laboratory of Cellular Biotechnology (RIBSP, RK). Semi-synthetic wall medium (SNM, RIBSP, RK) and DMEM (Capricorn Scientific GmbH, Ebsdorfergrund, Germany) supplemented with fetal bovine serum (FBS, Sigma, St. Louis, MO, USA) were used to grow cell cultures.

Knockout of the immunomodulatory genes LSDV005 and LSDV008 was carried out by deleting the corresponding sequences in the viral genome using the integration plasmids pIN-LSDV005Δ and pIN-LSDV008Δ [16]. The insertion of foreign sequences into the LSDV066 gene resulted in gene knockout due to a reading frame shift. Bovine interleukin-18 (bIL18) was used as a foreign antigen for insertion into the Lumpy skin disease virus genome. The bIL18 mRNA encoding sequence (GenBank ID: EU276078) was synthesized by Elabscience (Houston, TX, USA) as part of the pUC57 plasmid. The pIN-LSDV066-IL18 integration plasmid was used to knockout the LSDV066 gene [16].

Recombinant viruses were obtained by homologous recombination under conditions of transient dominant selection as described previously [17]. First, recombinants Atyrau-5, Atyrau-B, and Atyrau-J(IL-18) with knockout of one of the immunomodulatory genes LSDV005, LSDV008, and LSDV066, respectively, were obtained. Then, the bIL18 sequence was inserted into the thymidine kinase locus of Atyrau-5 and Atyrau-B viruses. As a result, recombinant viruses Atyrau-5J(IL-18) and Atyrau-BJ(IL-18), respectively, were obtained. Table 1 presents data on the recombinant viruses obtained and used in this study.

The growth curve of recombinant Lumpy skin disease virus (LSDV) strains was evaluated in LT and Vero cell. Six-well plates were seeded with LT or Vero cells in the appropriate complete medium containing 10% FBS. The plates were incubated until 90–100% confluency of the cell monolayer. Cells were infected at a multiplicity of infection of 0.01 TCID_50_/cell. Infected cells were incubated at 37 °C in a humidified atmosphere containing 5% CO_2_ for 144 h. Samples were collected every 12 h to assess viral infectivity. Cells were lysed by two freeze–thaw cycles. Viral titers were determined by microtitration assay in LT cell cultures. Infectivity was calculated according to the Reed and Muench method [18] and expressed as lg TCID_50_/mL. Final titration results were recorded on day 10 post-infection.

### 2.2. Immunoelectron Microscopy

A monolayer of LT cells was infected with the Atyrau-5J(IL18) virus at dose 0.1 TCID_50_/cell. Forty-eight hours after infection cells were mechanically collected from the walls of the flask. Then cells were sequentially fixed with 2% glutaraldehyde in cacodylate buffer and 2% osmium tetroxide in water. After washing in 0.1 M cacodylate buffer, the cells were treated in alcoholic solutions with a concentration of 50, 70, 80, 90, 96%, incubating for 10 min in each solution. Then the cells were incubated for 15 min in acetone and a mixture of acetone-resin (1:1). The cells were then transferred to resin and incubated overnight. A mixture of epoxy resins Epon 812 (Sigma, Steinheim, Germany) and Araldite M (Sigma, Steinheim, Germany) was used with the addition of DDSA (Sigma, Steinheim, Germany) sealant and DMP-30 (Sigma, Steinheim, Germany) accelerator in the following ratios: 20 mL Araldite M, 25 mL Epon 812, 60 mL DDSA, 2 mL DMP-30.

Ultrathin sections of cells were obtained on a microtome LKB ULTROTOME III 8800, Sweden using glass knives. Section thickness was determined by color. The silver-gray sections were 400–500 Å thick. Sections were caught on copper nets and dried on filter paper.

For immunodetection of bIL18, grids with ultrathin sections were sequentially placed on drops of blocking solution, mouse anti-bIL18 antibodies and protein A conjugated with colloidal gold. Between treatments, the grids were washed in phosphate buffer, pH 7.4. After incubation with gold, the grids were washed with distilled water (2–3 times, 15–20 min). Then grids were stained with 2% uranyl acetate in water for 30 min and 1% lead citrate for 1 min. After drying the grids on filter paper, an electron microscopic analysis of the studied samples was carried out on a transmission electron microscope JEM-100 CX-II JEOL, (JEOL Ltd., Tokyo, Japan) at an accelerating voltage of 80 kV and a magnification of 10,000×.

### 2.3. Western Blotting

Immunodetection of bovine IL18 expressed by recombinant Lumpy skin disease viruses was performed by Western blotting as described previously [19]. Mouse polyclonal anti-bovine IL18 antibodies were used for immunodetection of the target antigen and anti-β-actin antibodies (Thermo Fisher Scientific, Northern Ind. Zone, Kiriyat Shemona, Israel) as a loading control to confirm uniformity of sample loading.

### 2.4. Immunization of Mice

To assess the expression of bovine IL18 antigen by recombinant viruses, forty outbred white mice aged 5–7 weeks were used. The mice were randomly divided into four groups. The mice were immunized subcutaneously in the withers area twice with an interval of 21 days. Live viruses without any adjuvants were used for immunization at a dose of 6 lgTCID_50_/0.1 mL.

Fourteen days after the second injection, blood was taken from the tail vein of the mice to determine the humoral immune response using the ELISA method. Then the mice were killed by cervical dislocation, the spleen was removed to isolate splenocytes and evaluate cellular immunity.

The study was conducted according to the guidelines of the Declaration of Helsinki, and approved by the Committee on the Ethics of Animal Experiments of the Research Institute for Biological Safety Problems MH RK (Permit Number 11/2022, approved 15 November 2022).

### 2.5. ELISA

96-well plates were coated with bacterially expressed recombinant interleukin-18; 100 μL of carbonate-bicarbonate buffer containing 2 μg/mL of recombinant protein was added to the wells. The plates were incubated overnight at 4 °C. To remove unbound proteins, the plate wells were washed three times with TBS-T (300 μL/well) followed by blocking with 5% skim milk in TBS-T (200 μL/well). Sera from mice immunized with Lumpy skin disease viruses were diluted with TBS-T in a ratio of 1:8 and 100 μL (in duplicate) was added to the wells of the plate and incubated for 1 h at 37 °C. Then the plate wells were washed three times with TBS-T buffer (300 μL/well), and anti-mouse antibodies conjugated to alkaline phosphatase were added at a dilution of 1:5000 and incubated for 1 h at 37 °C. After washing three times, 100 μL of substrate for alkaline phosphatase (pNPP) was added to the wells of the plate and incubated for 30 min. The ELISA results were recorded using an ImmunoChem-2100 reader at a wavelength of 405/630 nm. Threshold values were determined using the mean optical density (OD) values of negative control sera plus two standard deviations (SD). Serum samples whose optical density exceeded the threshold value were considered positive.

### 2.6. Cytokine Secretion Assay

The spleens were removed from mice killed by cervical dislocation. The spleen was placed in a Petri dish. Using a syringe, 5–10 mL of DMEM was passed through the organ, causing cells to wash out into the medium. This action was repeated until the cells left the organ completely. The cell suspension was transferred into a 15 mL tube and centrifuged at 800× *g* for 3 min at room temperature. After destruction of red blood cells, splenocytes were washed twice with culture medium. The sediment was suspended in 3 mL of complete medium and the cell concentration was determined.

Splenocytes were induced using RPMI1640 medium, recombinant IL18 (10 μg/mL), Lumpy skin disease virus (1000 TCID_50_/mL), and concanavalin A (3 μg/mL). The inductors (500 µL) were added to the wells of a 24-well plate and preheated at 37 °C. A suspension of splenocytes was prepared at a concentration of 4 × 10^6^ cells/mL and 500 µL were added to the wells to the corresponding inductors. Two replicates were used for each variant. Cells with inducers were incubated for 72 h. Fine-Test kits (Wuhan Fine Biotech Co., Wuhan, China) were used to determine the concentrations of cytokines in the supernatant of the cell suspension by ELISA according to the manufacturer’s instructions.

### 2.7. Statistical Data Analysis

Statistical analysis of experimental data, plotting of graphs and diagrams were performed using Graphpad Prism version 6.0 (Graphpad Software Inc., San Diego, CA, USA) and Microsoft Excel 2021 MSO. The results of the quantitative analysis are presented as the mean ± standard error of the mean (SEM) of three independent experiments. The approaches used to analyze the obtained experimental data are indicated in the figure captions.

## 3. Results

### 3.1. Recombinant Lumpy Skin Disease Viruses: Generation and Characterization

As a result of the research, five recombinant viruses were obtained based on the parent virus Atyrau/KZ. The presence of deletions and insertions in the genomes of the recombinant viruses, as well as their genetic stability, was confirmed using PCR as described previously [16] (see Figures S1 and S2, Supplementary Data).

**Figure 1:**
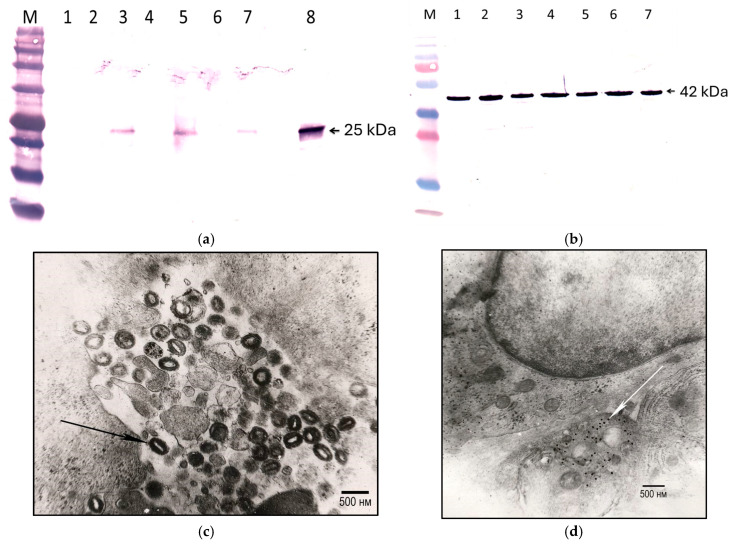
Immunodetection of bovine interleukin-18 expressed by recombinant Lumpy skin disease viruses. (**a,b**): gel loaded with 7 μL of lamb testicular (LT) cell lysates uninfected-mock (lane 1) and infected with recombinant viruses Atyrau/KZ (lane 2), Atyrau-J(IL-18) (lane 3); Atyrau-5 (lane 4); Atyrau-5J(IL-18) (lane 5); Atyrau-B (lane 6); Atyrau-BJ(IL-18) (lane 7); and 0.5 μg of purified bacterially expressed bovine interleukin-18 (lane 8). M—prestained protein standard (**a**-P7712, NEB, Ipswich, MA, USA, **b**-26619, Thermo Fisher Scientific, Vilnius, Lithuania). Serum from mice immunized with bacterially expressed bovine interleukin 18 (**a**) and anti-beta actin antibodies (Thermo Fisher Scientific, Northern Ind. Zone, Kiriyat Shemona, Israel) (**b**). Ultrathin sections of lamb cells infected with Atyrau-5 (**c**) and Atyrau-5J(IL18) (**d**) viruses after immunodetection. Black arrows indicate virions; white arrows indicate gold particles bound to foreign antigens.

It was found that recombinant Lumpy skin disease viruses with one or two knocked out genes remained genetically stable for ten consecutive passages in LT cell culture (Figures S1 and S2, Supplementary File S1). Expression of the inserted foreign gene (bovine IL18) also confirmed the genetic stability of the recombinant viruses. Bovine IL18 was detected by Western blotting in cell lysates (Figure 1a,b) and by immunoelectron microscopy in infected cells (Figure 1c,d). bIL18 expressed by recombinant Lumpy skin disease viruses, as well as the bacterially expressed control protein, specifically bound to anti-serum (Figure 1a). Electron microscopic studies of ultrathin sections of infected cells showed that bIL18 expressed by the recombinant virus Atyrau-5J(IL18) is localized in the cytosol of cells near the virions (Figure 1d).

### 3.2. Effect of Gene Knockout on the Replication Activity of Recombinant Viruses

**Figure 2:**
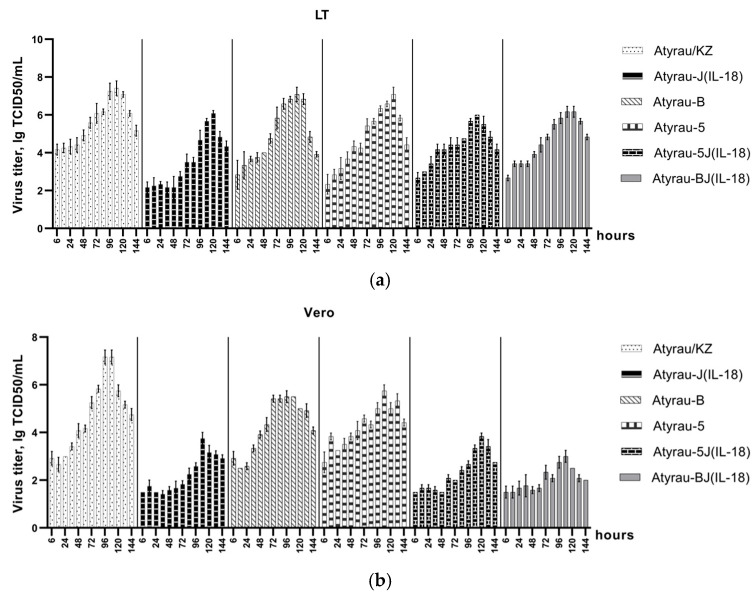
The kinetics of replication of Lumpy skin disease viruses (**a**) in lamb testicular (LT) and (**b**) in African green monkey kidney (Vero) cell cultures (explanations in the text).

The kinetics of replication of recombinant viruses were assessed in permissive lamb testis cells (LT) and non-permissive African green monkey kidney cells (Vero) (Figure 2).

On average, the maximum accumulation of the studied viruses was observed by 108 h of the infectious process. At the same time, in non-permissive Vero cells, the replication of the studied viruses was less productive, which was expressed in a decrease in the titers of infectious activity. It was found that the parental LSDV Atyrau/KZ replicated effectively in LT and Vero cell cultures, accumulating to 7.42 ± 0.18 and 7.17 ± 0.14 TCID_50_/mL, respectively (Figure 2, Supplementary File S2). Deletion of one of the immunomodulatory genes (LSDV005, LSDV008, LSDV066) influenced the replication of the recombinant viruses and led to a decrease in titers in both LT and Vero cells. The infectious activity titers of the recombinant viruses Atyrau-5, Atyrau-B and Atyrau-J(IL-18) in LT cells after 108 h were 6.58 ± 0.07, 7.08 ± 0.18 and 5.67 ± 0.07 TCID_50_/mL, respectively. In Vero cells, the infectious activity titers of the recombinant viruses with knockout of one immunomodulatory gene (Atyrau-5, Atyrau-B and Atyrau-J(IL-18)) were significantly lower and were 5.75 ± 0.12, 5.50 ± 0.00 and 3.75 ± 0.12 TCID_50_/mL, respectively.

**Figure 3:**
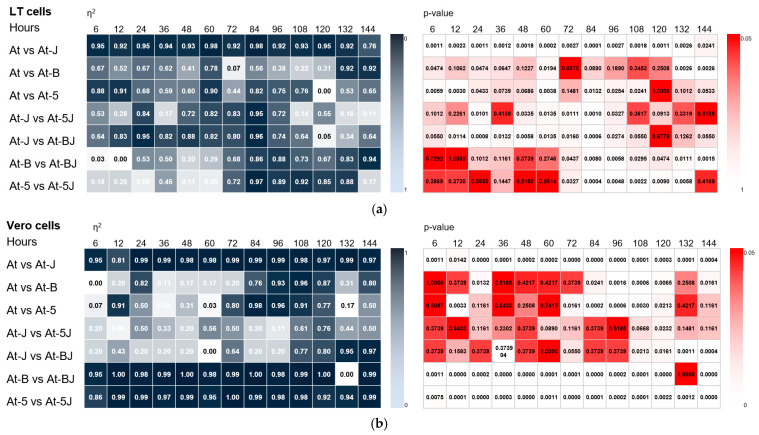
Analysis of the effect of knockout of immunomodulatory genes on the replication activity of recombinant Lumpy skin disease viruses. Viral replication activity was determined (**a**) in lamb testicular cells (LT) and (**b**) African green monkey kidney (Vero) cells. One-way ANOVA of the infectivity of viruses with knockout of one or two immunomodulatory genes. Statistical significance was defined at α ≤ 0.05. Effect sizes were calculated using eta-squared (η^2^) to estimate the proportion of variance explained by a factor. η^2^ values are presented as heat maps, where darker shades correspond to higher statistical significance. At—parent Lumpy skin disease virus Atyrau/KZ, At-J—recombinant Atyrau-J(IL18) virus, At-5—recombinant Atyrau-5 virus, At-5J—recombinant Atyrau-5J(IL18) virus, At-B—recombinant Atyrau-B virus, A-BJ—recombinant Atyrau-BJ(IL18) virus.

**Figure 4:**
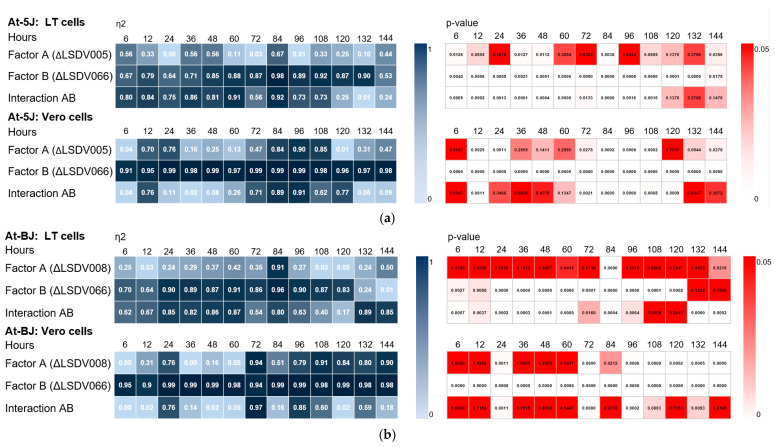
Analysis of the effect of knockout of immunomodulatory genes on the replication activity of recombinant Lumpy skin disease viruses. Two-way ANOVA was performed to evaluate the independent and combined effects of (**a**) LSDV005 (factor A) and LSDV066 (factor B) and (**b**) ΔLSDV008 (factor A) and LSDV066 (factor B) gene deletions on the replication of recombinant Lumpy skin disease viruses in LT and Vero cells. Heat maps display the effect size (η^2^, (**left**)) and corresponding *p*-values (**right**) for each factor and their interaction (AB). Statistical significance was defined at α = 0.05. Higher η^2^ values indicate a higher proportion of variance explained by the factor. Lighter shades in the *p*-value heat maps correspond to higher statistical significance. At-5J—recombinant Atyrau-5J(IL18) virus, A-BJ—recombinant Atyrau-BJ(IL18) virus, LT—lamb testicular cell.

A significant effect of LSDV005 gene knockouts on recombinant virus replication was observed at late stages of the infection process (84–108 h) in non-permissive cells (Figure 3b and Figure 4a. Supplementary Files S3 and S4). The mean η^2^ value was ≥0.8 at *p*-value < 0.05. The effect of LSDV008 gene knockout on replication of recombinant viruses was also detected only in non-permissive cells during the period from 84 to 144 h of viral infection (Figure 3b and Figure 4b. Supplementary Files S3 and S4). The mean η^2^ value was ≥0.8 at *p*-value < 0.05. Genotype-dependent differences were evident in Vero cells, which is likely due to the absence of specific immunomodulatory interactions that may be present in LT cells.

It was found that the disruption of the LSDV066 immunomodulatory gene has the greatest effect on LSDV replication in both permissive and non-permissive cells (Figure 3a,b. Supplementary File S3) compared to knockout of the LSDV005 or LSDV008 genes. This effect was particularly pronounced for recombinants replicating in non-permissive Vero cells (Figure 3b, Supplementary File S3). The mean η^2^ value was ≥0.90 with *p*-value < 0.01.

Our studies have shown that knockout of the immunomodulatory gene LSDV066 in the genomes of recombinant viruses Atyrau-5 and Atyrau-B leads to a significant change in the replicative activity in both permissive and non-permissive cells. Two-way ANOVA showed that the reduction in replicative activity of recombinant viruses with knockout of two immunomodulatory genes (LSDV005 and LSDV066 or LSDV008 and LSDV066) was largely due to the disruption of the LSDV066 gene (Figure 4a,b. Supplementary File S4).

Thus, the negative effect of LSDV066 disruption on recombinant viruses’ replication was observed in both single knockout and double knockout (LSDV005 and LSDV066 or LSDV008 and LSDV066). These data suggest that LSDV066 plays a critical role in ensuring efficient viral replication, potentially through modulating interactions with the host cell. These results are consistent with previous studies that have suggested *Capripoxvirus* immunomodulatory genes such as ORF066 as rational targets for reducing virulence [1].

### 3.3. Effect of Gene Knockout on the Immunogenicity of Recombinant Lumpy Skin Disease Viruses

To assess immunogenic activity, mice were immunized with recombinant viruses. Humoral and cellular immune responses were assessed.

#### 3.3.1. Humoral Immune Response

**Figure 5:**
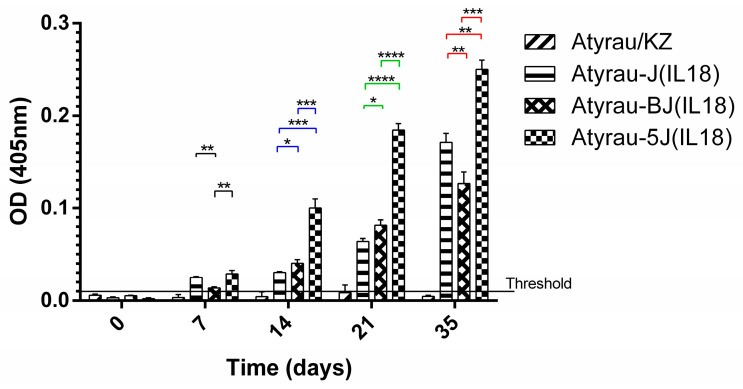
Immune response to foreign antigens expressed by recombinant viruses in mice after prime-boost immunization. Levels of specific antibodies to bovine interleukin-18 were measured by indirect ELISA. Statistical analysis was performed using ordinary one-way ANOVA followed by Tukey’s multiple comparisons test. Statistically significant differences between groups are indicated in black on day 7, blue on day 14, green on day 21, and red on day 35. *P*-values < 0.05 were considered significant. *—*p* < 0.05, **—*p* < 0.005, ***—*p* < 0.0005, ****—*p* < 0.00001.

The resulting recombinant viruses were evaluated for their ability to induce antibody production in mice. Analysis of the blood serum of mice immunized with recombinant viruses showed that all viruses expressing foreign antigens induce the production of antibodies (Figure 5). The highest titer of antibodies to the foreign antigen interleukin-18 was observed in the blood sera of mice immunized with a recombinant virus with a knockout of two genes in the combination LSDV005 and LSDV066.

#### 3.3.2. Cellular Immune Response

**Figure 6:**
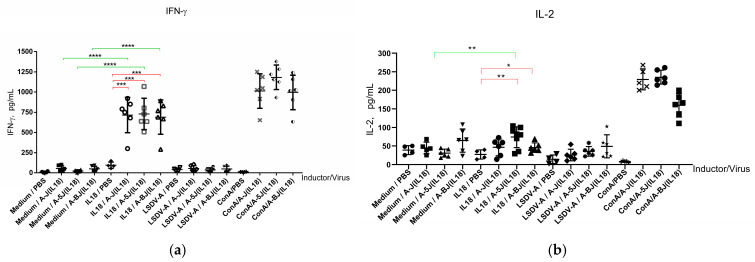
The level of cytokines produced by splenocytes of mice immunized with indicated Lumpy skin disease viruses or controls. (**a**) IFN-γ, (**b**) IL-2. The *x*-axis indicates the cytokine expression inductors used and the viruses used to immunize mice. Medium—RPMI1640 nutrient medium for cell cultures, PBS—phosphate-buffered saline solution, IL-18—recombinant bacterially expressed bovine interleukin-18, A-J(IL18)—recombinant Atyrau-J(IL18) virus, A-5J(IL18)—recombinant Atyrau-5J(IL18) virus, A-BJ(IL18)—recombinant Atyrau-BJ(IL18) virus, LSDV-A—parent Lumpy skin disease virus Atyrau/KZ. Statistical analysis was performed using ordinary one-way ANOVA followed by Tukey’s multiple comparisons test. Statistically significant within-group comparisons are indicated by red lines, and between-group comparisons are indicated by green lines. *p* values < 0.05 were considered significant. *—*p* <0.05, **—*p* < 0.005, ***—*p* < 0.0005, ****—*p* < 0.00001.

Cellular-mediated immune responses play an essential role in the defense against viruses. Cytokine expression levels were determined by ELISA (Figure 6).

Figure 6a shows that knockout of the LSDV005 and LSDV008 genes did not affect the production of interferon-γ by splenocytes of mice immunized with recombinant Atyrau-5J(IL18) and Atyrau-BJ(IL18) viruses after stimulation with interleukin-18 (an antigen expressed by recombinant Lumpy skin disease viruses). At the same time, deletion of the LSDV005 gene led to a significant (*p* < 0.01) increase in the level of interleukin-2 expression in splenocytes of mice immunized with the recombinant Atyrau-5J(IL18) virus.

## 4. Discussion

Rational design of effective vaccine vectors that provide a long-term immune response to expressed foreign antigens requires knowledge about the mechanisms of the vector’s influence on the formation of the immune response in the body. Immunomodulatory genes of the viral vector play an important role in this process. Since the current state of knowledge does not allow us to predict in advance the effects that will be achieved if one or more genes are disrupted, each emerging variant of the virus requires careful study.

Most of the studies involving the deletion of immunomodulatory genes have been performed on the Western Reserve strain of vaccinia virus, and the overall results showed that the deletion of many genes resulted in attenuation of the virus [13], but the effect on immunogenicity was variable. An innovative new approach to optimizing the vaccine vector based on poxviruses, by increasing immunogenicity of foreign antigens, is the combination of heterologous antigen insertion and immunomodulatory gene deletion [14].

An example of an attenuated vaccinia virus vector is the NYVAC strain obtained by deleting 18 genes from the Copenhagen vaccinia virus genome. Among them were 12 immunomodulatory genes [20]. Deletions of numerous genes resulted in the inability of the virus to replicate in most human and mammalian cells. However, NYVAC provides high levels of gene expression and induced antigen-specific immune responses to target proteins to animals and humans [21,22].

Studies examining the functions of capripoxvirus genes are sparse. Thus, an immunomodulatory gene, the knockout of which enhanced the immune response to a foreign antigen, was identified for the goat pox virus [23]. This protein functioned as an inhibitor of NF-κB activation and apoptosis and is similar to the N1L protein of vaccinia virus. Zhang et al. [23] using gene 135 as the insertion site, generated a recombinant strain AV41 expressing peste-des-petits ruminants (PPR) virus hemagglutinin, which elicited stronger neutralizing antibody responses than those generated using the traditional *tk* gene as the insertion site.

We characterized the replication of recombinant LSDVs with knockout of immunomodulatory genes in permissive and nonpermissive cells. Disruption of the LSDV066 gene encoding thymidine kinase resulted in a sharp decrease in the titer of viral infectious activity. This effect was characteristic of both viruses with knockout of LSDV066 alone and in combination with the LSDV005 or LSDV008 genes. Thymidine kinase is a key enzyme for viral DNA replication, which phosphorylates thymidine to dTMP. Despite this, the thymidine kinase gene is used as a target for insertion of foreign sequences into the *Orthopoxvirus* genome. Deletion of the gene results in a decrease in virus virulence [1]. Wallace and Viljoen in their studies established that thymidine kinase is an important component for the replication of Lumpy skin disease virus [24]. Our studies also confirm the importance of maintaining the integrity of this gene for efficient viral replication in vitro.

The effect of the LSDV005 and LSDV008 genes knockout on the replication of LSDVs was not as significant as knockout of the LSDV066 gene. However, differences were noted in the formation of an immune response to a model antigen bIL18 expressed by recombinant viruses with knockout of the LSDV005 and LSDV008 genes. As in most studies of the effectiveness of LSDV-based vaccine vectors, we used mice to assess the immune response [9,10].

It was found that the highest level of antibodies to the model antigen bIL18 was induced by the LSDV Atyrau-5J(IL18) with knockout of LSDV005. Antibodies play an important role in protecting against infection in a number of infectious diseases. Induction of a humoral response to the integrated antigen is one of the primary requirements for a vaccine vector.

The cell-mediated immune response also plays a key role in viral infections. Proinflammatory cytokines provide support and enhancement of cytotoxic T cells. Among them is interferon-γ, which plays an important role in protective immunity during viral infections [25]. Deletion of the immunomodulatory genes LSDV008 and LSDV005 did not affect the production of interferon-γ by mouse splenocytes. The LSDV008 gene encodes a soluble interferon-γ receptor-like protein. This protein inhibits the interaction between host interferon-γ and its receptor, preventing the antiviral effect of interferon-γ [26]. Studies by Kara et al. showed that knockout of the LSDV005 or LSDV008 genes does not result in loss of viral infectivity in cattle [27].

At the same time, deletion of the LSDV005 gene resulted in a reliable (*p* < 0.01) increase in the level of interleukin-2 expression by splenocytes of mice immunized with the recombinant Atyrau-5J(IL18) virus. The LSDV005 gene is expressed in the early stages of infection and acts like cellular interleukin-10 (IL10), influencing the formation of the immune response. By imitating the suppressive effect of host IL10 on Th1-mediated responses, it promotes evasion of the immune response [28]. It is likely that knockout of this gene contributed to a shift in the immune response towards the Th1 type, as evidenced by an increase in IL2 expression. IL2 is a characteristic cytokine of the Th1 immune response, stimulates the growth of T helper, cytotoxic and regulatory T cells, and also promotes the differentiation of T cells into effector T cells and memory T cells, which helps the body fight infection [29].

## 5. Conclusions

The study of the functions of immunomodulatory genes and their influence on the interaction of the vaccine vector with host cells is promising for the creation of highly effective polyvalent vector vaccines for animals. In this work, the effect of the immunomodulatory genes LSDV005, LSDV008 and LSDV066 of the Lumpy skin disease virus on the replicative activity of recombinants and the formation of an immune response to a foreign antigen was studied. The results of the studies showed that knockout of the LSDV005 gene contributed to the formation of a higher level of antibodies to a foreign antigen and induced the expression of the IL2 cytokine, characteristic of the Th-1 immune response. Preservation of the activity of thymidine kinase encoded by the LSDV066 gene is important for effective replication of LSDV. For the insertion of foreign sequences, it is optimal to use the intergenic space or other target genes.

## Figures and Tables

**Table 1:** Recombinant Lumpy skin disease viruses obtained in this study.

Virus Name	Genome Mutation		
	LSDV005 Gene Deletion	LSDV008 Gene Deletion	LSDV066 Gene IL-18 Insertion
Atyrau/KZ	−	−	−
Atyrau-5	+	−	−
Atyrau-B	−	+	−
Atyrau-J(IL-18)	−	−	+
Atyrau-5J(IL-18)	+	−	+
Atyrau-BJ(IL-18)	−	+	+

## Data Availability

The data presented in this study are available on request from the corresponding author.

## Supplementary Materials

The following supporting information can be downloaded at: https://www.mdpi.com/article/10.3390/v17101390/s1, Supplementary File S1: PCR analysis of recombinant viruses after 5 and 10 passages. Supplementary File S2: Biological activity of recombinant viruses during passaging in cell cultures. Supplementary File S3: One-Way ANOVA test and Heatmaps. Supplementary File S4: Two-Way ANOVA test and Heatmaps.

## References

[1] Tulman E.R., Afonso C.L., Lu Z., Zsak L., Sur J.H., Sandybaev N.T., Kerembekova U.Z., Zaitsev V.L., Kutish G.F., Rock D.L. (2002). The genomes of sheeppox and goatpox viruses. J. Virol..

[2] Tulman E.R., Afonso C.L., Lu Z., Zsak L., Kutish G.F., Rock D.L. (2001). Genome of lumpy skin disease virus. J. Virol..

[3] Moss B., Knipe D.M., Howley P.M. (2001). Poxviridae: The virus and their replication. Fields Virology.

[4] Haegeman A., De Leeuw I., Mostin L., Campe W.V., Aerts L., Venter E., Tuppurainen E., Saegerman C., De Clercq K. (2021). Comparative Evaluation of Lumpy Skin Disease Virus-Based Live Attenuated Vaccines. Vaccines.

[5] Aspden K., van Dijk A.A., Bingham J., Cox D., Passmore J.A., Williamson A.L. (2002). Immunogenicity of a recombinant lumpy skin disease virus (neethling vaccine strain) expressing the rabies virus glycoprotein in cattle. Vaccine.

[6] Wallace D.B., Mather A., Kara P.D., Naicker L., Mokoena N.B., Pretorius A., Nefefe T., Thema N., Babiuk S. (2020). Protection of Cattle Elicited Using a Bivalent Lumpy Skin Disease Virus-Vectored Recombinant Rift Valley Fever Vaccine. Front. Vet. Sci..

[7] Fakri F., Bamouh Z., Ghzal F., Baha W., Tadlaoui K., Fihri O.F., Chen W., Bu Z., Elharrak M. (2018). Comparative evaluation of three capripoxvirus-vectored peste des petits ruminants vaccines. Virology.

[8] Douglass N., Omar R., Munyanduki H., Suzuki A., de Moor W., Mutowembwa P., Pretorius A., Nefefe T., Schalkwyk A.V., Kara P. (2021). The Development of Dual Vaccines against Lumpy Skin Disease (LSD) and Bovine Ephemeral Fever (BEF). Vaccines.

[9] Shen Y.J., Shephard E., Douglass N., Johnston N., Adams C., Williamson C., Williamson A.L. (2011). A novel candidate HIV vaccine vector based on the replication deficient Capripoxvirus, Lumpy skin disease virus (LSDV). Virol. J..

[10] Aspden K., Passmore J.A., Tiedt F., Williamson A.L. (2003). Evaluation of lumpy skin disease virus, a capripoxvirus, as a replication-deficient vaccine vector. J. Gen. Virol..

[11] Haig D.M. (2001). Subversion and piracy: DNA viruses and immune evasion. Res. Vet. Sci..

[12] Nash P., Barrett J., Cao J.X., Hota-Mitchell S., Lalani A.S., Everett H., Xu X.M., Robichaud J., Hnatiuk S., Ainslie C. (1999). Immunomodulation by viruses: The myxoma virus story. Immunol. Rev..

[13] Smith G.L., Benfield C.T.O., Maluquer de Motes C., Mazzon M., Ember S.W.J., Ferguson B.J., Sumner R.P. (2013). Vaccinia virus immune evasion: Mechanisms, virulence and immunogenicity. J. Gen. Virol..

[14] García-Arriaza J., Esteban M. (2014). Enhancing poxvirus vectors vaccine immunogenicity. Hum. Vaccines Immunother..

[15] Orynbayev M.B., Nissanova R.K., Khairullin B.M., Issimov A., Zakarya K.D., Sultankulova K.T., Kutumbetov L.B., Tulendibayev A.B., Myrzakhmetova B.S., Burashev E.D. (2021). Lumpy skin disease in Kazakhstan. Trop. Anim. Health Prod..

[16] Chervyakova O., Issabek A., Sultankulova K., Bopi A., Kozhabergenov N., Omarova Z., Tulendibayev A., Aubakir N., Orynbayev M. (2022). Lumpy Skin Disease Virus with Four Knocked Out Genes Was Attenuated In Vivo and Protects Cattle from Infection. Vaccines.

[17] Chervyakova O., Tailakova E., Kozhabergenov N., Sadikaliyeva S., Sultankulova K., Zakarya K., Maksyutov R.A., Strochkov V., Sandybayev N. (2021). Engineering of Recombinant Sheep Pox Viruses Expressing Foreign Antigens. Microorganisms.

[18] Reed L.J., Muench H.A. (1938). Simple Method of Estimating Fifty Per Cent Endpoints. Am. J. Epidemiol..

[19] Chervyakova O.V., Zaitsev V.L., Iskakov B.K., Tailakova E.T., Strochkov V.M., Sultankulova K.T., Sandybayev N.T., Stanbekova G.E., Beisenov D.K., Abduraimov Y.O. (2016). Recombinant Sheep Pox Virus Proteins Elicit Neutralizing Antibodies. Viruses.

[20] Tartaglia J., Cox W.I., Taylor J., Perkus M., Riviere M., Meignier B., Paoletti E. (1992). Highly attenuated poxvirus vectors. AIDS Res. Hum. Retroviruses.

[21] Gomez C.E., Najera J.L., Jimenez E.P., Jimenez V., Wagner R., Graf M., Frachette M.J., Liljestrom P., Pantaleo G., Esteban M. (2007). Head-to-head comparison on the immunogenicity of two HIV/AIDS vaccine candidates based on the attenuated poxvirus strains MVA and NYVAC co-expressing in a single locus the HIV-1BX08 gp120 and HIV-1(IIIB) Gag-Pol-Nef proteins of clade B. Vaccine.

[22] Mooij P., Balla-Jhagjhoorsingh S.S., Beenhakker N., van Haaften P., Baak I., Nieuwenhuis I.G., Heidari S., Wolf H., Frachette M.J., Bieler K. (2009). Comparison of human and rhesus macaque T-cell responses elicited by boosting with NYVAC encoding human immunodeficiency virus type 1 clade C immunogens. J. Virol..

[23] Zhang M., Sun Y., Chen W., Bu Z. (2018). The 135 Gene of Goatpox Virus Encodes an Inhibitor of NF-κB and Apoptosis and May Serve as an Improved Insertion Site To Generate Vectored Live Vaccine. J. Virol..

[24] Wallace D.B., Viljoen G.J. (2002). Importance of thymidine kinase activity for normal growth of lumpy skin disease virus (SA-Neethling). Arch Virol.

[25] Mueller S.N., Rouse B.T. (2009). Immune responses to viruses. Clin. Immunol..

[26] Johnston J.B., McFadden G. (2003). Poxvirus immunomodulatory strategies: Current perspectives. J. Virol..

[27] Kara P.D., Mather A.S., Pretorius A., Chetty T., Babiuk S., Wallace D.B. (2018). Characterisation of putative immunomodulatory gene knockouts of lumpy skin disease virus in cattle towards an improved vaccine. Vaccine.

[28] Imlach W., McCaughan C.A., Mercer A.A., Haig D., Fleming S.B. (2002). Orf virus-encoded interleukin-10 stimulates the proliferation of murine mast cells and inhibits cytokine synthesis in murine peritoneal macrophages. J. Gen. Virol..

[29] Liao W., Lin J.X., Leonard W.J. (2011). IL-2 family cytokines: New insights into the complex roles of IL-2 as a broad regulator of T helper cell differentiation. Curr. Opin. Immunol..

