# Supplementary material for: Assessment of Biological Properties of Recombinant Lumpy Skin Disease Viruses with Deletions of Immunomodulatory Genes
Source: Viruses. 2025 Oct 19;17(10):1390. doi: 10.3390/v17101390 (PMC12567934; doi:10.3390/v17101390)
Supplement: Supplementary file 1 [file viruses-17-01390-s001.zip › Supplementary File S1.pptx]

## Slide 1
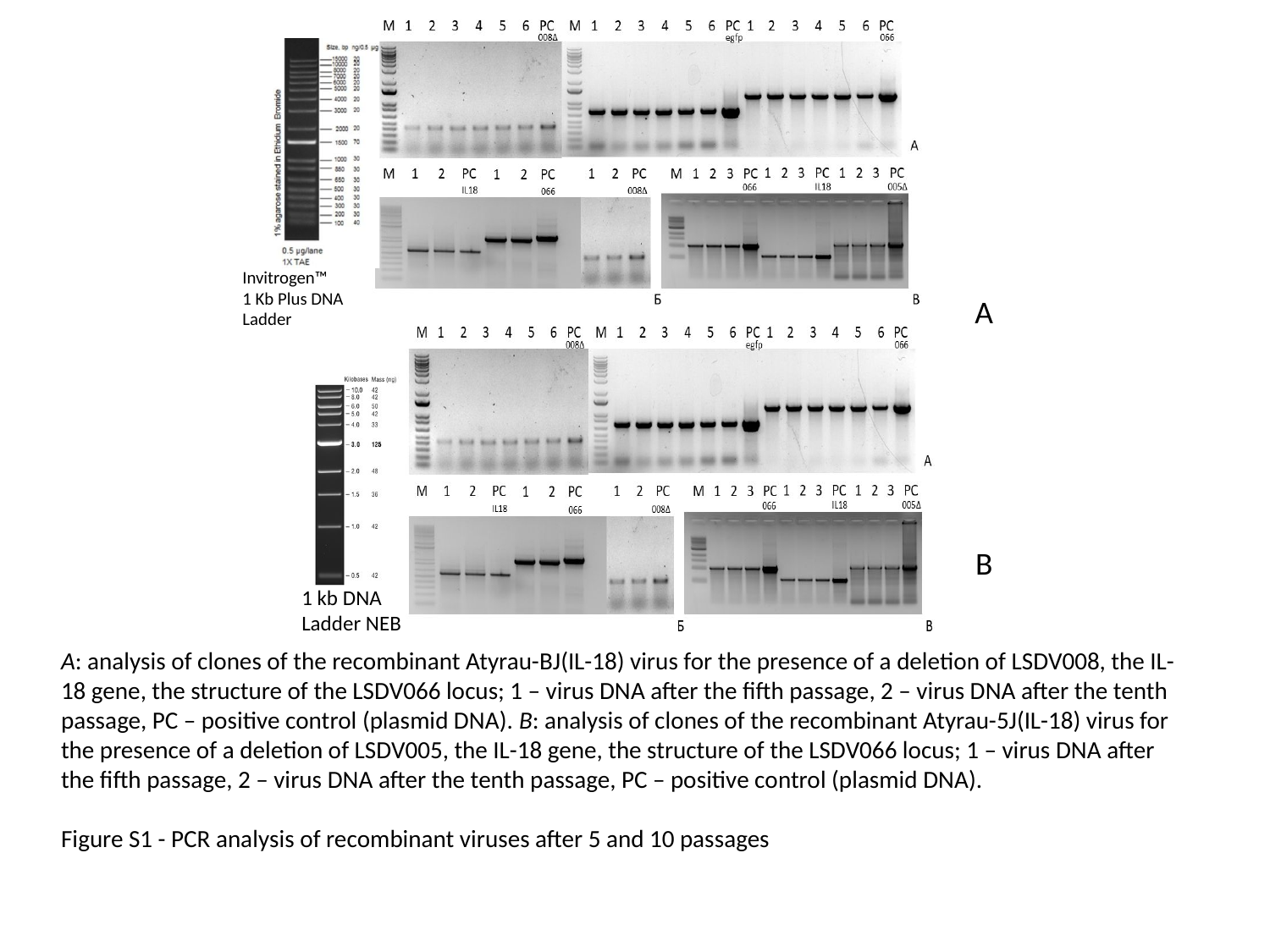

Invitrogen™
1 Kb Plus DNA Ladder
A
B
1 kb DNA Ladder NEB
A: analysis of clones of the recombinant Atyrau-BJ(IL-18) virus for the presence of a deletion of LSDV008, the IL-18 gene, the structure of the LSDV066 locus; 1 – virus DNA after the fifth passage, 2 – virus DNA after the tenth passage, PC – positive control (plasmid DNA). B: analysis of clones of the recombinant Atyrau-5J(IL-18) virus for the presence of a deletion of LSDV005, the IL-18 gene, the structure of the LSDV066 locus; 1 – virus DNA after the fifth passage, 2 – virus DNA after the tenth passage, PC – positive control (plasmid DNA).
Figure S1 - PCR analysis of recombinant viruses after 5 and 10 passages

## Slide 2
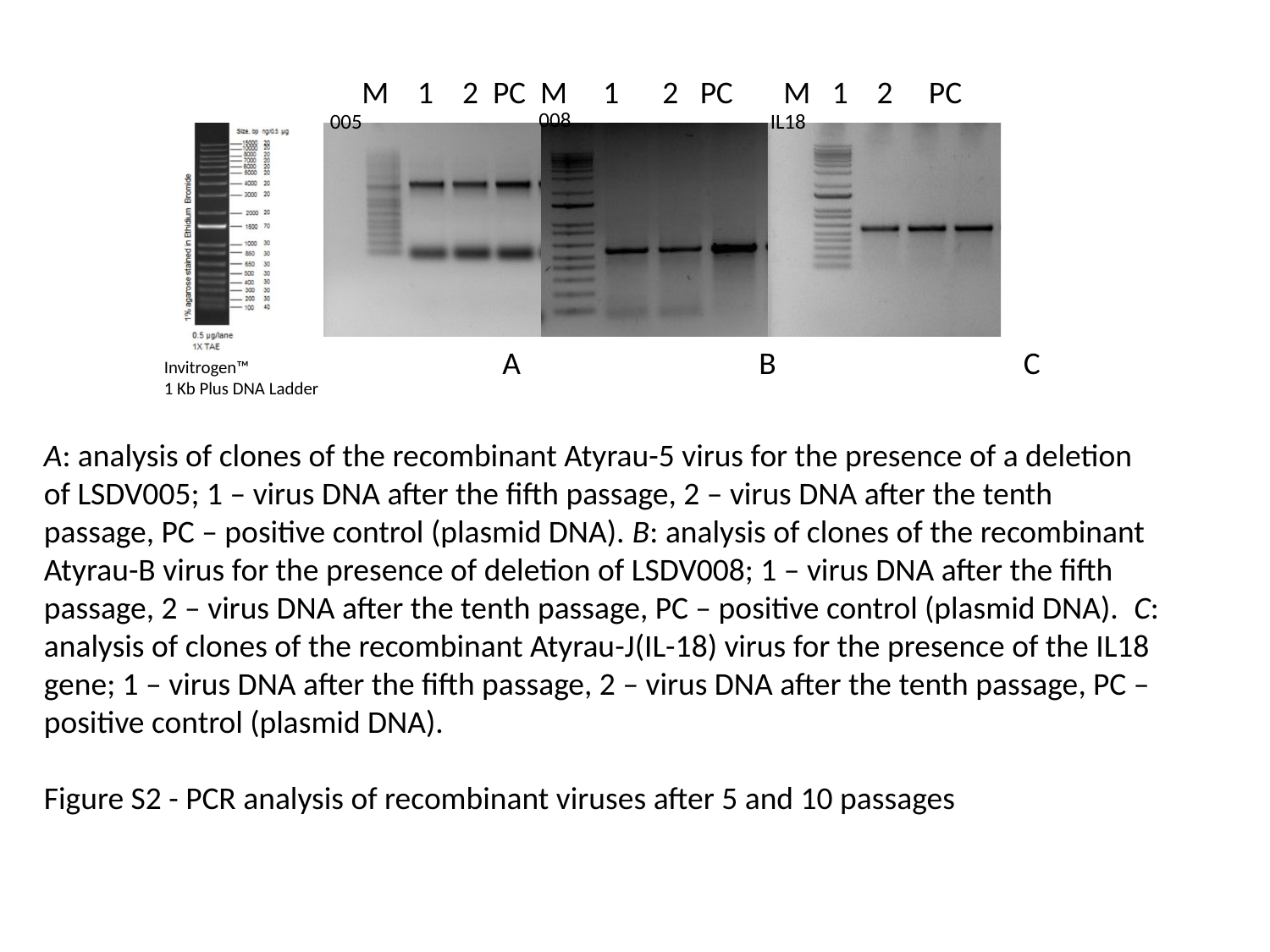

M 1 2 PC M 1 2 PC M 1 2 PC
A
C
B
008
005
IL18
Invitrogen™
1 Kb Plus DNA Ladder
A: analysis of clones of the recombinant Atyrau-5 virus for the presence of a deletion of LSDV005; 1 – virus DNA after the fifth passage, 2 – virus DNA after the tenth passage, PC – positive control (plasmid DNA). B: analysis of clones of the recombinant Atyrau-B virus for the presence of deletion of LSDV008; 1 – virus DNA after the fifth passage, 2 – virus DNA after the tenth passage, PC – positive control (plasmid DNA). C: analysis of clones of the recombinant Atyrau-J(IL-18) virus for the presence of the IL18 gene; 1 – virus DNA after the fifth passage, 2 – virus DNA after the tenth passage, PC – positive control (plasmid DNA).
Figure S2 - PCR analysis of recombinant viruses after 5 and 10 passages
